# An In Vitro Study of the Effect of *Viburnum opulus* Extracts on Key Processes in the Development of Staphylococcal Infections

**DOI:** 10.3390/molecules26061758

**Published:** 2021-03-21

**Authors:** Urszula Wójcik-Bojek, Joanna Rywaniak, Przemysław Bernat, Anna Podsędek, Dominika Kajszczak, Beata Sadowska

**Affiliations:** 1 Department of Immunology and Infectious Biology, Institute of Microbiology, Biotechnology and Immunology, Faculty of Biology and Environmental Protection, University of Lodz, Banacha 12/16, 90-237 Lodz, Poland; urszula.wojcik@biol.uni.lodz.pl (U.W.-B.); joanna.rywaniak@biol.uni.lodz.pl (J.R.); 2 Department of Industrial Microbiology and Biotechnology, Faculty of Biology and Environmental Protection, Institute of Microbiology, Biotechnology and Immunology, University of Lodz, Banacha 12/16, 90-237 Lodz, Poland; przemyslaw.bernat@biol.uni.lodz.pl; 3 Faculty of Biotechnology and Food Sciences, Institute of Molecular and Industrial Biotechnology, Lodz University of Technology, Stefanowskiego 4/10, 90-924 Lodz, Poland; anna.podsedek@p.lodz.pl (A.P.); dominika.kajszczak@dokt.p.lodz.pl (D.K.)

**Keywords:** biofilm, cell membrane lipids, infections, plant extracts, prevention, sortase A, *Staphylococcus aureus*, staphylococcal protein A, *Viburnum opulus*

## Abstract

*Staphylococcus aureus* is still one of the leading causes of both hospital- and community-acquired infections. Due to the very high percentage of drug-resistant strains, the participation of drug-tolerant biofilms in pathological changes, and thus the limited number of effective antibiotics, there is an urgent need to search for alternative methods of prevention or treatment for *S. aureus* infections. In the present study, biochemically characterized (HPLC/UPLC–QTOF–MS) acetonic, ethanolic, and water extracts from fruits and bark of *Viburnum opulus* L. were tested in vitro as diet additives that potentially prevent staphylococcal infections. The impacts of *V. opulus* extracts on sortase A (SrtA) activity (Fluorimetric Assay), staphylococcal protein A (SpA) expression (FITC-labelled specific antibodies), the lipid composition of bacterial cell membranes (LC-MS/MS, GC/MS), and biofilm formation (LIVE/DEAD BacLight) were assessed. The cytotoxicity of *V. opulus* extracts to the human fibroblast line HFF-1 was also tested (MTT reduction). *V. opulus* extracts strongly inhibited SrtA activity and SpA expression, caused modifications of *S. aureus* cell membrane, limited biofilm formation by staphylococci, and were non-cytotoxic. Therefore, they have pro-health potential. Nevertheless, their usefulness as diet supplements that are beneficial for the prevention of staphylococcal infections should be confirmed in animal models in the future.

## 1. Introduction

Despite the progress in microbiology, diagnostics, and medicine, *Staphylococcus aureus* is still one of the leading causes of human infections, including skin and soft tissue infections as well as many invasive, life-threatening contagions such as bacteremia; organ ulcers; bone, joint, and bone marrow infections; infective endocarditis; sepsis or toxic shock syndrome [1,2,3,4,5,6]. Being a component of the human microbiota, a broad panel of virulence factors that allow these bacteria efficient tissue colonization, as well as the avoidance, impairment, and modulation of the host immune response, underlie the success of *S. aureus* as a pathogen [5,6,7,8]. Most infections start from microbial adhesion to the host cells and elements of the extracellular matrix (proteoglycans, glycoproteins, tissue fibers) or to inserted/implanted biomaterials (e.g., intravascular catheters, dental implants, prosthetic valves, orthopaedic implants). The next step is the aggregation and multiplication of microorganisms, which very often leads to biofilm formation in host tissues [9,10,11,12,13]. Therefore, staphylococci possess a particularly wide repertoire of adhesins, such as microbial surface components recognizing adhesive matrix molecules (MSCRAMMs), which mediate staphylococcal attachment, tissue colonization and biofilm formation. Among them, clumping factors A and B (ClfA, ClfB), fibronectin-binding proteins A and B (FnBPA, FnBPB), collagen-binding protein (Cna), vitronectin-binding protein (Vbp), serine-aspartate repeat family proteins (SdrC, SdrD, and SdrE), staphylococcal protein A (SpA), *S. aureus* surface protein G (SasG), and biofilm-associated protein (Bap) have been mentioned. Some secretory factors (Eap, Efb, vWbp) also participate in staphylococcal attachment [6,11,14,15]. *S. aureus* adhesins also play roles in host cell invasion (intracellular persistence), cell activation (blood platelet aggregation and degranulation, cytokine and reactive oxygen species release by immunocompetent cells, extracellular adhesive molecule expression) and in the development of inflammation [6,15,16]. Thus, not only the beginning but also the course of *S. aureus* infections strongly depends on the expression of adhesins and the adhesive properties of these bacteria.

The treatment of staphylococcal infections, especially biofilm-associated infections, is particularly difficult due to the high percentage of multidrug-resistant strains, as well as the biofilm structure and physiology of these bacteria, which give them increased tolerance to environmental conditions, including antibiotics [12,17,18]. Therefore, the idea of blocking the initial adhesion and biofilm formation of *S. aureus* using plant-origin preparations containing biologically active compounds seems to be a reasonable strategy for the prevention of these infections. Many pro-health properties of plant-derived products, including antioxidative, antiallergic, anti-inflammatory, anticancer, antiatherosclerotic, antimicrobial, or topically anesthetic effects are described in the literature [19,20,21,22]. With respect to prevention or therapy of the infections, antimicrobial activity is the most attractive mode. However, aside from a direct biostatic/biocidal effect, the impacts on the virulence factors or pathogenic behaviors of microorganisms (e.g., adhesion, biofilm formation, cell invasion, quorum-sensing) can be considered with regard to the activity of plant preparations. Rasamiravaka et al. [23] demonstrated that crude ethyl acetate and dichloromethane extracts from *Platostoma rotundifolium* as well as those from isolated terpenoids disrupted *Pseudomonas aeruginosa* biofilm without affecting bacterial viability. The inhibitory effect of the terpenoids on some virulence factors of these bacteria, as well as on the expression of quorum-sensing-regulated and -regulatory genes was observed [23]. Howell et al. [24] showed that proanthocyanidin-containing powder from cranberries (*Vaccinium macrocarpon* Ait.) inhibited the adhesion of P-fimbriated uropathogenic *E. coli* to epithelial T24 cells. Moreover, urine collected from volunteers following the consumption of cranberry powder was shown to possess the same effect, although this was clearly dose-dependent [24]. In our previous studies, promising data were provided that pointed to a limitation of *S. aureus* aggregation in the plasma, adhesion to abiotic or extracellular matrix protein-coated surfaces, and biofilm formation caused by polyphenol-rich *Leonurus cardiaca* extract [25]; phenolic and nonpolar fractions separated from *Elaeagnus rhamnoides* leaf, twig, and fruit extracts [26]; *Pulmonaria officinalis* extract [27]. Based on data in the literature, one of the most promising mechanisms of antistaphylococcal action is the inhibition of *S. aureus* sortase A (SrtA) activity, which may affect the expression of a wide range of staphylococcal adhesins. The sortase enzymes are highly conserved membrane transpeptidases that catalyze the incorporation of cell surface proteins into the microbial cell wall. SrtA covalently binds surface staphylococcal proteins containing the C-terminal pentapeptide LPXTG motif (Leu-Pro-X-Thr-Gly, with X standing for any amino acid except for cysteine) to peptidoglycan precursors by cleaving the amide bond between threonine and glycine [28,29,30]. Since the most prominent staphylococcal surface adhesins are MSCRAMMs containing the LPXTG motif, SrtA represents a desirable target for drug development [30,31,32,33].

The subjects of the present research were the extracts obtained from *Viburnum opulus* L., known also as European guelder, European cranberry bush, water elder, rose elder, Rose Ebru, cherry-wood, crampbark, snowball tree, or gilaburu, which is a deciduous shrub that is native to Europe, north Africa, and north and central Asia. It belongs to the Adoxaceae family (formerly Caprifoliaceae) [20,34,35]. The biological activity of *V. opulus* preparations seems to be very broad, including antioxidative effects, direct antimicrobial activity (biostatic effect against many pathogenic Gram-positive and Gram-negative bacteria, as well as yeast from *Candida* sp.), hypoglycemic activity, hypotensive effect, the prevention of lipid uptake and adipogenesis, an anti-inflammatory effect, anticancer potential, osteogenic activity, uterine relaxant and antispasmodic properties, a diuretic effect, and preventive activity against crystal deposition and stone formation [35,36,37,38,39,40,41,42,43,44]. Thus, *V. opulus* is widely used in natural medicine for the relief of asthma, cold, fever, cough, nervousness, rheumatoid diseases, diabetic, menstrual cramps, uterine and urinary tract infections, and to treat high blood pressure [20,45]. Various parts of this plant are used, primarily the fruits (*Viburni fructus recens*) and bark (*Viburni opuli cortex*), which are believed to have anti-inflammatory, diaphoretic, and diuretic properties [46,47].

The aim of this study was to test the antistaphylococcal activity of originally obtained, well biochemically characterized acetonic (a), ethanolic (e), and water (w) extracts from fruits and bark of *Viburnum opulus* L. (VF and VB, respectively) to determine for the first time their effects on the adhesiveness and biofilm formation of *S. aureus*. The impacts of these extracts on staphylococcal virulence factors, such as SrtA activity, SpA surface expression, and the composition of bacterial cell membranes, that can determine the development of biofilm-associated infections, were assessed. A relationship between the observed mode of action of plant preparations and the actual adhesive properties of *S. aureus* cells was considered based on biofilm formation by staphylococci pre-exposed to the extracts. Assuming the final use of *V. opulus* extracts in vivo as pro-health dietary additives, their cytotoxicity to the human foreskin fibroblast line HFF-1 was first tested.

## 2. Results

### 2.1. Biochemical Characterization of V. opulus Fruit and Bark Extracts

The chemical compositions of crude extracts obtained from *V. opulus* fruit and bark are presented in Table 1. The total protein content varied from 8.64 to 16.13 mg/g, the sugar content varied from 162.38 to 674.69 mg/g, and the organic acid content was in the range of 4.26–86.36 mg/g. It was noted that *V. opulus* fruit extracts were superior to bark extracts in terms of sugar and organic acid contents. On the other hand, bark extracts were more abundant in phenolic compounds, including flavanols and proanthocyanidins. The total content of phenolics, determined by a spectrophotometric method with the Folin–Ciocalteu reagent, varied from 50.64 to 254.97 mg/g, the content of flavanols estimated with the vanillin reagent was from 11.82 to 97.79 mg/g, and the proanthocyanidins level was from 5.81 to 63.38 mg/g. Quantitatively, the main components of the fruit extracts were sugars, followed by organic acids and phenolic compounds, while phenolic compounds and sugars were dominant in the bark extracts. Of the three solvents used for extraction in this study, water showed the highest affinity for proteins, irrespective of plant materials, and for flavanols in the case of bark. The highest sugar and organic acid contents was found in the *V. opulus* extracts separated from both fruit and bark with 70% ethanol. The acetonic fruit and bark extracts were the richest in total phenolic compounds and proanthocyanidins. Quantitative analysis of *V. opulus* fruit and bark extracts using the UPLC method showed that the fruit extracts were dominated by hydroxycinnamic acids (60.37–65.64 mg/g), which accounted for almost 83% of the total content of the determined phenolic groups. In turn, in bark extracts and flavanols (119.13–132.15 mg/g) accounted for an average of 74% of the total phenol content determined by the UPLC method. For comparison, flavonols had the lowest concentration in fruit extracts, and flavalignans had the lowest concentration in bark extracts. Qualitative analysis of the *V. opulus* fruit and bark extracts by the UPLC-QTOF-MS technique revealed that the phenolic compound composition differed notably. Twenty-one phenolic compounds were detected in fruit extracts and 15 in bark extracts (Table S1 and Figure S1 in Supplementary Materials).

### 2.2. Cytotoxicity of V. opulus Fruit and Bark Extracts

**Figure 1:**
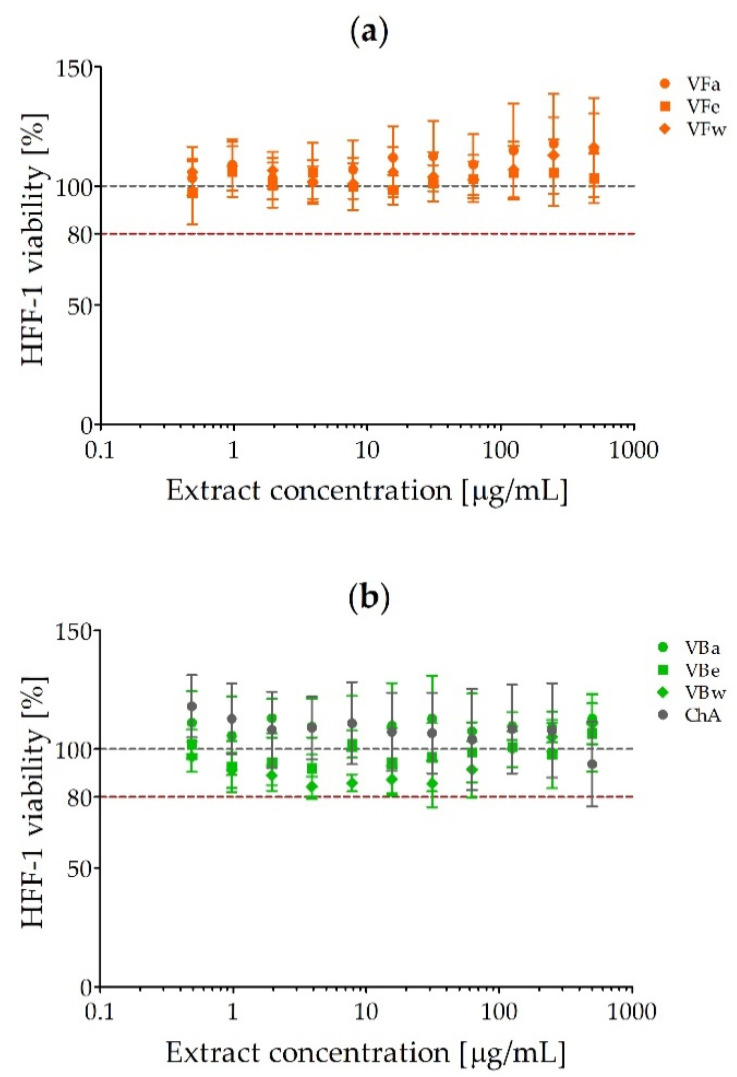
The cytotoxic activity of *V. opulus* extracts on the human foreskin fibroblast line HFF-1: (**a**) *V. opulus* fruit extracts (VF); (**b**) *V. opulus* bark extracts (VB); a/e/w—acetonic/ethanolic/water extract; ChA—chlorogenic acid.

The HFF-1 cells were exposed to *V. opulus* fruit and bark extracts or chlorogenic acid as a reference compound used at a concentration range of 0.49–500 µg/mL for 24 h. The results are given as percentages of cell viability in comparison with untreated control cells (100% viability) and are presented in Figure 1. It was demonstrated that none of the preparations tested possessed cytotoxic activity up to the concentration of 500 µg/mL. The viability of HFF-1 cells did not fall below 80%. Lower cell viability (84.33 ± 4.1%) was observed after exposure to water extract from *V. opulus* bark (VBw) at a concentration of 3.9 µg/mL (Figure 1b). Interestingly, the use of this extract at the highest concentration (500 µg/mL), as well as acetonic and water extracts from the fruits (VFa/w, Figure 1a) and acetonic extract from *V. opulus* bark (VBa, Figure 1b), seemed to slightly support fibroblast growth.

### 2.3. Minimal Inhibitory/Bactericidal Concentration (MIC/MBC) of V. opulus Extract against S. aureus

The biostatic and biocidal activity of *V. opulus* extract against *S. aureus* ATCC 29213 was assessed using the broth microdilution method. The obtained results are presented in Table 2. Acetonic extract from *V. opulus* bark (VBa) expressed the strongest antistaphylococcal activity with MIC = MBC = 1000 µg/mL. Thus, for the study, subinhibitory concentrations of the extracts were used (100 and 500 µg/mL) so as not to disturb microbial growth.

### 2.4. The Effect of V. opulus Extracts on SrtA Activity

**Figure 2:**
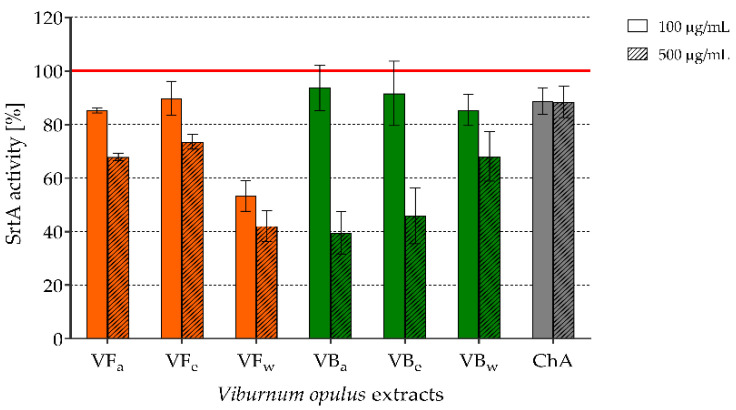
The effect of *V. opulus* extracts on staphylococcal sortase A (SrtA) activity after 30 min incubation with the substrate; VF—*V. opulus* fruit extract; VB—*V. opulus* bark extract; a/e/w—acetonic/ethanolic/water extract; ChA—chlorogenic acid.

Staphylococcal sortase A (SrtA) activity was evaluated outside bacterial cells using the SensoLyte 520 Sortase A Activity Assay Fluorimetric Kit. SrtA was exposed to *V. opulus* extracts or ChA at concentrations of 100 and 500 µg/mL. The results were collected after 30 min and 60 min of incubation of treated and control (untreated) SrtA with the substrate and are presented in Figure 2. The results are given as the percentage of SrtA activity calculated from relative fluorescence units (RFU) of test wells compared with the positive control (untreated SrtA), which was considered to represent 100% activity. All of the tested preparations inhibited SrtA activity. However, water extract from *V. opulus* fruits (VFw) showed one of the strongest effects and this was independent of concentration (46.7% and 57.9% of inhibition after exposure to 100 and 500 µg/mL, respectively; 30 min incubation with the substrate). Acetonic and ethanolic *V. opulus* bark extracts (VBa and VBe) possessed similar very strong inhibitory activity against SrtA, but this was limited to the higher concentrations (500 µg/mL) of these extracts (60.5% and 54.2% inhibition caused by VBa and VBe, respectively; 30 min incubation with the substrate). The prolonged 60 min SrtA incubation with the substrate did not change the observed effects (data not shown). Interestingly, chlorogenic acid alone had weaker inhibitory activity than *V. opulus* extracts.

### 2.5. Impact of V. opulus Extracts on SpA Expression

**Figure 3:**
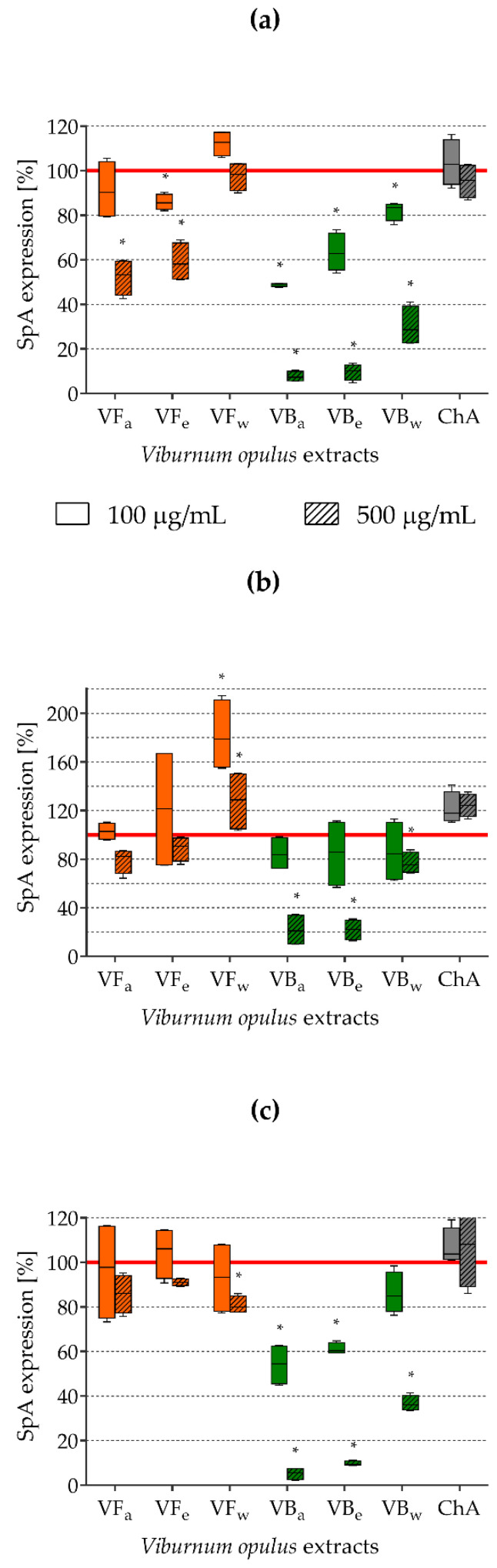
The effects of *V. opulus* extracts on staphylococcal protein A (SpA) expression on the bacterial cell surface: (**a**) *S. aureus* ATCC 29213; (**b**) *S. aureus* ATCC 43300; (**c**) *S. aureus* H9; VF—*V. opulus* fruit extract; VB—*V. opulus* bark extract; a/e/w—acetonic/ethanolic/water extract; ChA—chlorogenic acid; * statistically significant differences compared to the control (*p* ≤ 0.05).

To determine the effect of the tested extracts on SrtA activity in living bacterial cells, the expression of SpA (staphylococcal surface protein anchored to peptidoglycan by sortase) was tested using FITC-labelled goat polyclonal anti-SpA antibodies. Based on SpA-related fluorescence, the percentages of protein A expression on *S. aureus* cells exposed to *V. opulus* extracts or ChA compared with that the control (untreated) cells, taken as possessing 100% SpA expression, were calculated. The obtained results are presented in Figure 3. Irrespective of the staphylococcal strain tested, *V. opulus* bark extracts (VB) were the most effective for the inhibition of SpA expression. All types of VB extract used at both concentrations (100 and 500 µg/mL) almost always significantly decreased protein A expression, but higher concentrations of VBa and VBe were extremely active, causing 92.3% and 90.3% inhibition of SpA expression, respectively, on *S. aureus* ATCC 29213 cells (Figure 3a), 78.3% and 78.0% inhibition on *S. aureus* ATCC 43300 cells (Figure 3b), and 94.9% and 90.3% inhibition on *S. aureus* H9 cells (Figure 3c). Quite strong inhibitory activity was also noted for acetonic and ethanolic *V. opulus* fruit extracts (VFa and VFe), although this was limited to higher concentrations and mainly occurred with *S. aureus* ATCC 29213 (Figure 3a).

### 2.6. The Effects of the Tested Extracts on the Compositions of Phospholipids and Fatty Acids in Staphylococcal Membranes

**Figure 4:**
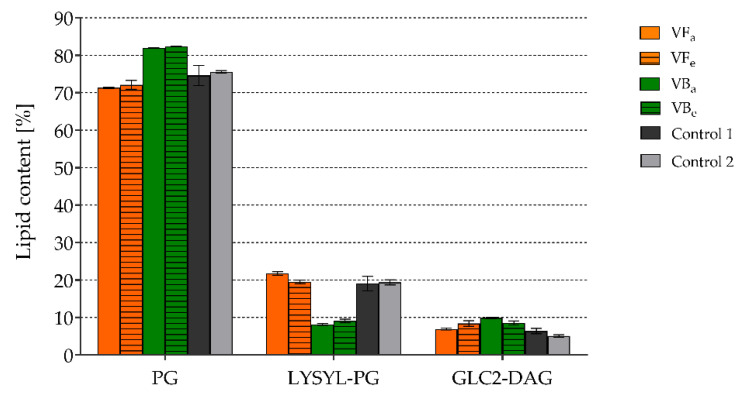
The effects of *V. opulus* extracts on the contents of the main classes of lipids in the cell membrane of *S. aureus* ATCC 43300; PG—phosphatidylglycerols; LYSYL-PG—lysyl-phosphatidylglycerols; GLC2-DAG—diglucosyldiacylglycerols; VF—*V. opulus* fruit extract; VB—*V. opulus* bark extract; a/e—acetonic/ethanolic extract; Control 1—bacteria in medium alone; Control 2—bacteria in medium containing 2.5% ethanol.

The impacts of *V. opulus* extracts on neutral lipid, phospholipid, and fatty acid types and their contents in the staphylococcal membrane were tested using LC-MS/MS and GC/MS techniques. Phosphatidylglycerols (PGs) were found to be the predominant lipid type in both *S. aureus* ATCC 43300 control and extract-treated cells. The PG content was from 71.33 ± 0.21% to 82.36 ± 0.06% (Figure 4). Among other lipids, lysyl-phosphatidylglycerols (LYSYL-PGs) and diglucosyldiacylglycerols (GLC2-DAGs) were also detected; however, their concentrations were 8.15 ± 0.22–21.75 ± 0.45% and 5.06 ± 0.35–9.90 ± 0.17%, respectively (Figure 4). Comparing control and extract-treated cells, clear differences were observed only for *S. aureus* exposed to *V. opulus* bark extracts (VBa and VBe). Interestingly, the PG and GLC2-DAG contents increased in the membranes of staphylococci exposed to VBa and VBe in comparison to control cells, while, at the same time, the concentration of LYSYL-PG was significantly reduced (Figure 4). The analysis of the changes in content of specific types of lipids and fatty acids are presented in Figure S2 and Figure S3, respectively, in Supplementary Materials.

### 2.7. Assessment of Biofilm Formation by Staphylococci Pre-exposed to V. opulus Extracts

**Figure 5:**
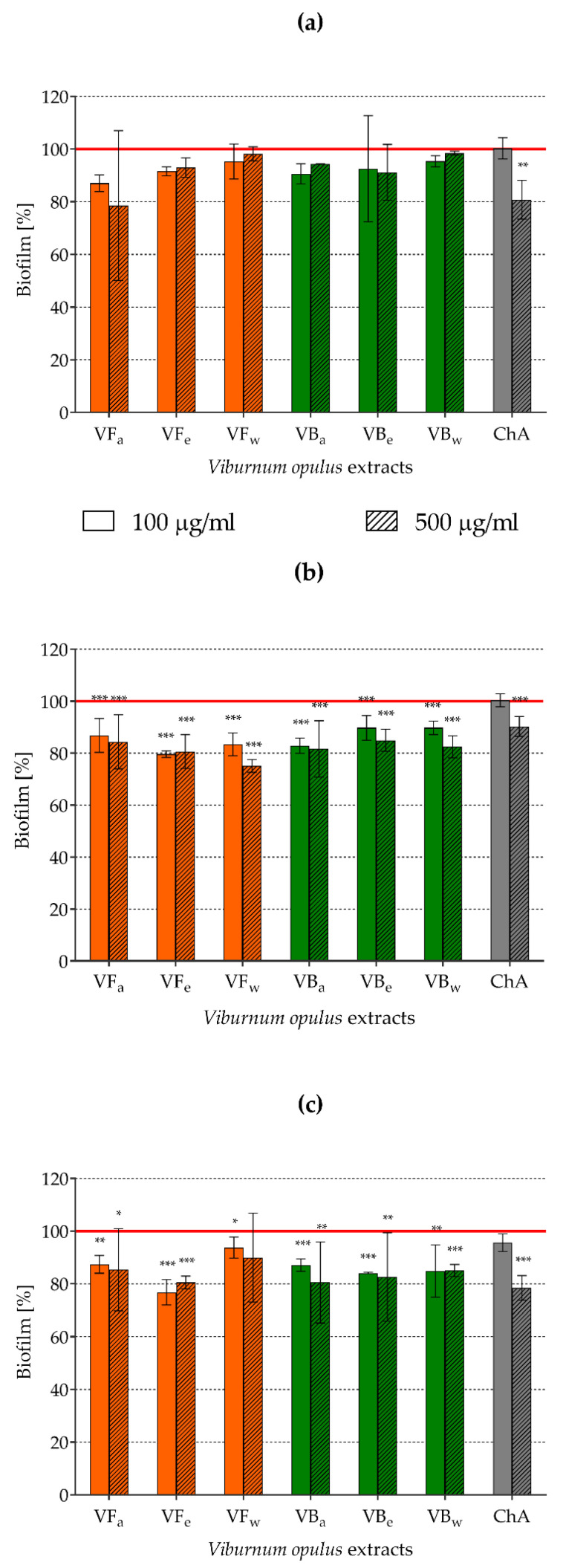
The effects of *V. opulus* extracts on the ability of staphylococci to form biofilm: (**a**) *S. aureus* ATCC 29213; (**b**) *S. aureus* ATCC 43300; (**c**) *S. aureus* H9; VF—*V. opulus* fruit extract; VB—*V. opulus* bark extract; a/e/w—acetonic/ethanolic/water extract; ChA—chlorogenic acid; statistically significant differences compared with the control: *p ≤ 0.05, ** *p* ≤ 0.01, *** *p* ≤ 0.001.

Attempts to limit staphylococcal adhesive properties using *V. opulus* extracts influencing SrtA activity and MSCRAMMs expression should be reflected in the process of biofilm formation. Therefore, biofilm biomass accumulation on abiotic polystyrene plates by staphylococci pre-exposed to *V. opulus* extracts was tested fluoromerically using the LIVE/DEAD BacLight Bacterial Viability Kit. As demonstrated in Figure 5, biofilm formation by *S. aureus* pre-exposed to all types of tested extracts used at both lower (100 µg/mL) and higher concentrations (500 µg/mL) as well as by chlorogenic acid at a higher concentration was inhibited. The observed effects were statistically significant for *S. aureus* ATCC 43300 (Figure 5b) and *S. aureus* H9 (Figure 5c) but not for *S. aureus* ATCC 29213 (Figure 5a). VFe seemed to be the most potent biofilm inhibitor, decreasing *S. aureus* ATCC 43300 biofilm biomass by 20.4 ± 1.3% (*p* = 0.000016) and 19.4 ± 6.5% (*p* = 0.00004), and *S. aureus* H9 biofilm biomass by 23.2 ± 4.8% (*p* = 0.000311) and 19.5 ± 2.4% (*p* = 0.000311) when it was used at 100 and 500 µg/mL, respectively.

## 3. Discussion

In the present study, the effects of originally obtained acetonic, ethanolic, and water extracts from *Viburnum opulus* fruits and bark on *S. aureus* virulence factors determining the development of staphylococcal biofilm-associated infections were investigated to establish the anti-infective potential of these extracts. The activity of *V. opulus*-derived products results mainly from the presence of phenolic compounds [20]. As reported by several authors, hydroxybenzoic and hydroxycinnamic acids (mainly chlorogenic, gallic and ferulic acid), flavanols (catechin, epicatechin, procyanidin), flavonols (quercetin and isorhamnetin glycosides), and anthocyanins (mainly cyanidin 3-glucoside, cyanidin 3-arabinosyl-glucoside, cyanidin 3-rutinoside, and cyanidin 3-sambubioside) have been identified in *V. opulus* fruits and fruit juice [35,48,49,50,51]. The qualitative composition of the extracts analyzed in the present study depended on the morphological part of the plant used for the extraction process, while the quantitative composition also depended on the type of extraction solvent used. The crude extracts tested here were highly heterogeneous and were composed of macronutrients (sugars, proteins, and organic acids) and a large variety of phytochemicals, such as phenolic compounds. The bark extracts exceeded the fruit extracts approximately three times, seven times, and more than six times in terms of the total phenolics, flavanols, and proanthocyanidins, respectively (Table 1). Eleven hydroxycinnamic acids, four flavonols, three flavanols, and three flavalignans were identified in *V. opulus* fruit extracts. As a comparison, five hydroxycinnamic acids, eight flavanols, and two flavalignans were identified in *V. opulus* bark extracts. A previous study [51] showed that hydroxycinnamic acids are dominant in *V. opulus* fruits, while flavanols are dominant in bark. The results obtained for the extracts tested in the present study are consistent with the cited data. Hydroxycinnamic acids comprised almost 83% of the total content of the different phenolic groups in fruit extracts, while flavanols accounted for an average of 74% of the total phenols determined by the UPLC method in bark extracts. Chlorogenic acid, as the compound present in all tested extracts, was used as a reference compound in the present study. Data on the compositions of individual phenolic compounds are greatly important, because their structures significantly affect their properties. All *V. opulus* extracts tested were not cytotoxic to human fibroblasts up to a concentration of 500 µg/mL. However, considering the usually weak bioavailability of phenolic compounds after oral administration and their poor tissue distribution, we decided to test the effects when the extracts were used at a low concentration of 100 µg/mL. It is also worth emphasizing that the MIC/MBC of these extracts against staphylococci did not go below 1000 µg/mL (Table 2); thus, the concentrations chosen for the research did not disturb bacterial growth.

As we explained before, both the initiation and course of staphylococcal infections strongly depend on the adhesive properties of these bacteria. Therefore, we assumed that the inhibition of the expression of bacterial adhesins could limit staphylococcal adhesion and biofilm formation and thus prevent the development of *S. aureus* infections. Sortase A (SrtA), a membrane-localized cysteine transpeptidase, which catalyzes the covalent bonding of staphylococcal surface adhesive proteins, called MSCRAMMs, to peptidoglycan, seems to be a good candidate for use as an antimicrobial drug target [7,33]. It has been demonstrated that a lack of SrtA causes the defective anchoring of about 20 staphylococcal surface proteins (including ClfA and SpA), which may reduce *S. aureus* binding to the von Willebrand factor (vWF), the vWF-binding protein (vWbp), and finally to endothelial cells through protein bridges [7]. Based on data presented in the literature, in which some secondary plant metabolites, including flavonols (e.g., morin, myricetin, and quercetin) as well as chalcones (intermediate products in flavonoid biosynthesis), are shown to exhibit strong sortase inhibitory activity [30,31], we hypothesized that *V. opulus* extracts, which are rich in phenolic compounds, may also have such an effect. Here, it was demonstrated that all of the tested extracts inhibited SrtA activity, but the strongest effect (above 50% of inhibition) was associated with the water extract from *V. opulus* fruits (VFw) as well as acetonic and ethanolic bark extracts (VBa and VBe) used at a concentration of 500 µg/mL (Figure 2). The use of chlorogenic acid alone as a reference compound was associated with inhibitory activity, although this was not as strong as that of the extracts, which indicates the possibility of a synergistic effect among their various compounds. Wang et al. [52] showed that chlorogenic acid binds to the active site of SrtA and thus blocks the attachment of the substrates containing the LPXTG motif. Five other hydroxycinnamic acids—caffeic acid, neochlorogenic caid, cryptochlorogenic acid, and ferulic and isoferulic acids—were also identified as potent inhibitors of this Gram-positive bacteria transpeptidase. Chlorogenic acid exhibited the best sortase-inhibitory activity with an IC_50_ of 34 µg/mL, followed by caffeic acid (IC_50_ 155 µg/mL), ferulic acid (IC_50_ 257 µg/mL), and neo- and cryptochlorogenic acids with IC_50_ values of 300 µg/mL [53]. All three chlorogenic acids were identified in both *V. opulus* fruit and bark extracts, while caffeic acid was only present in fruit extracts (Table S1 in Supplementary Materials). The inhibitory activity of fruit extracts against SrtA could have also resulted from the presence of flavonols, which were not found in bark extracts. According to Kang et al. [31], quercetin reduced the activity of SrtA by half at a concentration of 52.70 µM. Four quercetin glycosides were identified in all *V. opulus* fruit extracts. Of course, the addition of a sugar molecule to aglycone can influence the inhibitory activity of quercetin. However, Liu et al. [32] observed that quercitrin (quercetin 3-rhamnoside), present in *V. opulus* fruit extracts, could inhibit the catalytic activity of SrtA by binding directly to the active region of the enzyme.

Since the impact of *V. opulus* extracts on SrtA activity was tested on commercially available free enzyme, to test the effect on real cell-associated enzyme we also studied the expression of staphylococcal protein A (SpA) on the surface of *S. aureus* cells pre-exposed to the tested extracts. SpA contains the LPXTG motif; thus, its surface expression in metabolically active bacteria depends mainly on SrtA activity [29]. Of course, it is possible that compounds that are able to interfere with protein synthesis (e.g., antibiotics such as macrolides, tetracyclines, and aminoglycosides) [54] and the general metabolic activity of bacteria (biostatic compounds) may also limit the surface expression of bacterial proteins. However, in a preliminary study, we determined the minimum inhibitory/biocidal/fungicidal concentrations (MIC/MBC/MFC) of the tested *V. opulus* extracts against a wide range of Gram-positive and Gram-negative bacteria, as well as fungi, and their MIC/MBC against *S. aureus* did not go below 1 mg/mL (Table 2). Thus, the sub-inhibitory concentrations of *V. opulus* extracts used in the present research avoided any metabolic influences. The results obtained for SpA expression on the surface of staphylococcal cells corresponded well with free SrtA activity. We demonstrated that all types of VB extract (especially VBa and VBe) significantly reduced SpA expression on the surfaces of all three tested *S. aureus* strains (reference ATCC 29213, ATCC 43300, and clinical H9). The percentage of inhibition of SpA expression achieved was 78.0–94.9% for VB extracts used at a concentration of 500 µg/mL (Figure 3). The obtained results are relevant in the context of staphylococcal infections, since SpA participates not only in the adhesion of *S. aureus* to surfaces and host cells, aggregation and biofilm formation, it is also involved in the evasion of host defense mechanisms. SpA is able to bind to immunoglobulins (nonspecific reaction), vWF, and complement proteins, and through binding to cellular receptors such as FcγR, TNFR1, and GPIbα, as well as the activation of MAPK signaling, SpA induces the production of pro-inflammatory cytokines and participates in the development of pathological changes, e.g., during infective endocarditis, chronic wound infections, or osteomyelitis [29,55,56,57]. Moreover, its ability to bind Fcγ of IgG (type 1, 2, 4) and the V_H_3 heavy chains (Fab region) in IgG, IgM, or IgA plays an important role in host immune response evasion by blocking so-called opsonophagocytosis and a complement activation, as well as by stimulating the clonal expansion of lymphocytes B (Spa acts as superantigen), leading to their apoptosis [29,58]. Thus, the impressive inhibition of SpA expression on the surface of *S. aureus* cells demonstrated here using *V. opulus* extracts seems to be a promising way to prevent staphylococcal infections. Interestingly, chlorogenic acid used alone did not exhibit such inhibitory activity, which corresponds well with its much weaker impact on SrtA activity than the effect demonstrated for *V. opulus* extracts. ChA in the literature is presented as one of the main polyphenol compounds in the human diet. A great number of pro-health activities of ChA, including antioxidant, anti-inflammatory, antilipidemic, antidiabetic, antihypertensive, anticancer, antineurodegenerative, and antimicrobial effects have been described based on both in vitro and in vivo studies [59]. ChA antimicrobial activity, the most important, due to the purpose of this study, is very diverse with MIC values against *S. aureus* ranging from 0.016 to even 10 mg/mL, depending on the tested strain [59,60]. In the present research, the MIC and MBC values of ChA were above 2 mg/mL. Considering all of our results, it can be assumed that the antimicrobial activity of ChA is a consequence of its direct biostatic/biocidal effects, rather than its inhibitory impacts on microbial virulence factors, such as enzymatic activity or adhesin expression. The exact mechanism of ChA antimicrobial activity is not fully understood, but the inhibition of *S. aureus* proliferation and damage to the bacterial cell membrane followed by its increased permeability leading to microbial cell death are indicated as being the most likely modes of ChA action [59,61].

Bacterial membranes are the structures responsible for cell homeostasis, interaction with external factors and participation in microbial response to environmental changes; thus, they cannot be stable structures. Their lipid composition is constantly being modified depending on the living conditions, such as temperature, osmolarity or pH [62,63]. There is no doubt that modifications to the membrane composition and, thus, cell surface stabilization, membrane-associated protein topology, surface hydrophobicity and charge, and finally bacterial cell resistance [62,63,64] play an important role in microbial adhesion and biofilm formation [25]. Moreover, SrtA is a membrane-localized enzyme, so these changes may also determine its expression and activity. Therefore, the effects of *V. opulus* extracts used at 500 µg/mL on the profile of lipids and fatty acids in the *S. aureus* ATCC 43300 cell membrane were assessed using qualitative and quantitative tests. According to literature data, phosphatidylglycerols (PG) are predominant in *S. aureus* membranes; however, other phospholipids, such as lysyl-phosphatidylglycerols (LYSYL-PG) and diacylglycerols, as well as other glycosylated or conjugated lipids, such as diglucosyldiacylglycerols (GLC2-DAG) and monoglucosyldiacylglycerols, are also present [62,63]. Our results confirm this, since the PG content in both control and extract-treated staphylococcal cells was above 70%, while the content of LYSYL-PG and GLC2-DAG did not exceed 22% and 10%, respectively. By analyzing the impacts of *V. opulus* extracts on the general composition of the staphylococcal membrane, clear differences were only observed for *S. aureus* exposed to bark extracts (VBa and VBe). Both extracts increased PG and GLC2-DAG contents and caused a significant reduction in the LYSYL-PG content, compared with that in the control cells. PG is a precursor of LYSYL-PG, and, in a reaction mediated by a membrane protein called the multiple peptide resistance factor F (MprF), an aminoacyl group from lysine-tRNA is transferred to PG [62,63,64]. This reaction stabilizes the microbial membrane and modulates its charge, since anionic PG is converted to cationic LYSYL-PG. The described modification is recognized as one of the staphylococcal virulence factors that prevents membrane perturbation in the presence of cationic antimicrobials, such as antimicrobial peptides; thus, it is an important component of human and animal innate immunity or cationic antibiotics (e.g., daptomycin) [25,63,64,65]. In this study, we observed an increased content of PG but not LYSYL-PG in the membranes of staphylococci exposed to VBa and VBe, which may indicate the ability of these extracts to block a transformation of PG to LYSYL-PG. Since the single-nucleotide polymorphisms in *mprF* correlated with the changes in the LYSYL-PG profile were demonstrated for clinical *S. aureus* strains exposed to daptomycin [64], the influence of *V. opulus* extracts on MprF activity could be a possible mechanism responsible for the inhibitory effect observed in our study. Although this hypothesis needs to be confirmed in future, *V. opulus* bark extracts, in contrast to the previously tested *Leonurus cardiac* extract [25], seem to have additional promise as preparations to prevent the activation of some mechanisms of staphylococcal resistance.

We assumed that exposure to *V. opulus* fruits and bark extracts that influence SrtA activity, MSCRAMMs expression, and the lipid composition of *S. aureus* cell membranes would change the adhesive properties of those bacteria and, thus, the process of biofilm formation. Indeed, differences in biofilm biomass between control and extract-exposed *S. aureus* were observed, but the significant inhibitory effect mainly concerned *S. aureus* ATCC 43300 and did not exceed 24%. This means that staphylococci, despite their exposure to *V. opulus* preparations disturbing some mechanisms responsible for bacterial adhesion, are still able to form a biofilm. Staphylococci are known to be extremely efficient at aggregation and biofilm formation, in which many different factors participate. The general steps of building these complex structures are similar in all bacteria, including staphylococci, and comprise microbial adhesion (classic settled biofilms) or cell aggregation (free-floating biofilm aggregates), multiplication, microcolony formation and extracellular polymeric substances (EPS) production, and finally, biofilm maturation (phenotypic and functional cell differentiation) and dispersion (detachment of single cells or small aggregates) [9,12,13]. The initial adhesion of bacteria mediated by unspecific physico-chemical interactions, such as electrostatic bonds, hydrophobic interactions, or van der Waals forces, is then strengthened by specific adhesins. Staphylococci possess a wide range of surface adhesins, including the previously described MSCRAMM proteins that are covalently linked to peptidoglycan subunits thanks to SrtA activity; nevertheless, polysaccharide intercellular adhesin (PIA), being a homopolymer of partially deacetylated β-1,6-linked *N*-acetyl-glucosamine (PNAG), plays a pivotal role in cell-to-cell interactions, leading to the accumulation of staphylococcal biomass. PIA/PNAG is encoded by the intercellular adhesion operon *ica*, which contains *icaADBC* genes [12,54,66,67]. Thus, the inhibition of SrtA activity limiting the expression of surface adhesive proteins by *V. opulus* extracts demonstrated in this study may not be as strongly reflected in biofilm formation by staphylococci, as expected. Moreover, *S. aureus* strains that do not possess the *ica* operon are also able to form a biofilm. It has been revealed that some components or products of staphylococcal cells, such as teichoic acids, adhesive proteins (mainly SasG and Bap), extracellular DNA (eDNA), β-hemolysin, or even bacterial metabolites may participate in bacterial adhesion, forming a framework that stabilizes staphylococcal biofilm and also replace PIA/PNAG in its role as a mediator of intercellular interactions [12,54,66,68,69]. Thus, *V. opulus* fruit and bark extracts may limit biofilm formation by staphylococci, but the effect is strongly strain-dependent. Moreover, full inhibition of the development of these structures cannot be assumed using *V. opulus* extracts as the only preventive preparations. Since these conclusions were drawn on the basis of in vitro research, in vivo studies on animal models of staphylococcal infections with *V. opulus* extracts as dietary supplements should be done in the future.

## 4. Materials and Methods

### 4.1. Plant Material Preparation and Chemical Analysis

#### 4.1.1. Standards and Reagents

Hypergrade acetonitrile (Merck, Darmstadt, Germany) and formic acid (Sigma, Steinheim, Germany) were used for LC-MS. Reference compounds were obtained from Sigma-Aldrich (Steinheim, Germany) ((+)-catechin, caffeic acid, (−)-epicatechin, gallic acid, quercetin 3-rhamnoside, and quercetin 3-rutinoside), Extrasynthese (Lyon, France) (chlorogenic acid and quercetin 3-glucoside), and Phytolab (Vestenbergsgreuth, Germany) (neochlorogenic acid, cryptochlorogenic acid, 3,5-dicaffeoylquinic acid, procyanidin B1 and B2, and procyanidin C1). Ultrapure water (Simplicity^TM^ Water Purification System, Millipore, Marlborough, MA, USA) was used to prepare all solutions.

#### 4.1.2. Extract Preparation

Commercially available dried bark and fruits of *V. opulus* were purchased from “Farm Vit” (Szczecin, Poland) and “Natura Wita Ltd.” (Kopernia, Poland), respectively. Powdered plant materials (coffee grinder) were extracted with 70% acetone or 70% ethanol (1:20, *w*/*v*) on a magnetic stirrer for 3 h at room temperature. Then, the mixtures were incubated for 18 h at room temperature in the dark followed by the re-extraction on a magnetic stirrer for 3 h. After centrifugation at 5000 rpm for 10 min (MPW-351R, MPW Med. Instruments, Warszawa, Poland) the supernatants were evaporated at 40 °C (vacuum rotary evaporator RII, Büchi, Switzerland) in order to remove organic solvents. The aqueous phases were lyophilized to obtain the ethanolic extract and acetonic extract. Aqueous extracts of *V. opulus* bark and fruit were obtained by mixing powdered plant materials (10 g) with 200 mL of boiling water. Then, the mixture was simmered for 5 min and kept at room temperature for 15 min. The supernatants obtained after centrifugation were concentrated and then lyophilized. As a result of the described procedure, six extracts were obtained, including three from bark (VBa, VBe, VBw) and three from fruit (VFa, VFe, VFw). The abbreviations (a), (e), and (w) denote acetonic, ethanolic, and water extracts, respectively. For chemical analysis, stock solution of each extract was prepared at a concentration of 25 mg/mL in water, followed by centrifugation before analysis.

#### 4.1.3. Analysis of Chemical Composition of Extracts

The crude protein content was analysed by the Kjeldhal method with a conversion factor of 6.25. Total sugar and organic acid contents in the extracts were determined by the HPLC system with a refractive index (RI) detector and a photodiode array detector (210 nm) connected in series (Waters Corp., Milford, MA, USA), as described previously [51]. The total phenolic content of the extracts was determined spectrophotometrically with Folin–Ciocalteau reagent and expressed as milligrams of gallic acid equivalents per gram of extract (mg GAE/g). The total proanthocyanidin content was determined after acid depolymerisation in butanol–HCl solution with the corresponding anthocyanidins and is expressed as cyanidin equivalents per gram of extract (CYE/g). The analytical procedures for determining the contents of total phenolic compounds and proanthocyanidins were described in previous work [51]. The total flavanol content was estimated using the vanillin–sulfuric acid method, and results are expressed as milligrams of (+)-catechin equivalents per gram of extract (CE/g) [70].

#### 4.1.4. Analysis of Phenolic Compounds Using Ultra-Performance Liquid Chromatography–Quadruple–Time of Flight Mass Spectrometry (UPLC–QTOF–MS)

Phenolic compounds were identified using the Acquity ultraperformance liquid chromatography (UPLC) system coupled with a quadruple-time of flight mass spectrometry (Q-TOF-MS) instrument (Waters Corp., Milford, MA, USA) equipped with an electrospray ionization (ESI) source. Separation of individual phenolics was carried out using a Acquity UPLC^R^ HSS T3 C18 column (150 × 2.1 mm^2^, 1.8 µm; Corp., Milford, MA, USA) at 30 °C according to the method presented by Zakłos-Szyda et al. [44] with some modifications. The mobile phase was a mixture of 0.1% formic acid (A) and acetonitrile (B). The gradient program was as follows: initial conditions—99% (A), 12 min 65% (A), 12.5 min 0% (A), 13.5 min 99% (A). The flow rate was 0.45 mL/min, and the injection volume was 5 µL. The mass spectrometer was operated in negative mode for a mass range of 150–1500 Da, the fixed source temperature was 100 °C, the desolvation temperature was 250 °C, the desolvation gas flow was 600 L/h, the cone voltage was 45 V, the capillary voltage was 2.0 kV, and the collision energy was 50 V. Leucine enkephalin was used as a lock mass. The instrument was controlled by Mass-Lynx^TM^ V 4.1 software. Phenolic compounds were identified using their UV-Vis characteristics. MS and MS^2^ properties were determined using data gathered in house and from the literature. Based on the qualitative analysis of phenolic compounds, the total contents of flavanols, flavonols, flavalignans, hydroxycinnamic acids and iridoids were calculated. The contents are expressed in mg/g of extract for equivalents of (+)- catechin, quercetin 3-glucoside, cinchonine, chlorogenic acid, and quercetin 3-rutinoside, respectively.

### 4.2. Stock Solutions for Biological Tests

Stock solutions of *V. opulus* extracts (10 mg/mL or 50 mg/mL) were prepared in sterile-filtered water (the extracts from fruits, water extract from bark) or 50% ethanol (ethanolic and acetonic extracts from bark) and kept frozen at −20 °C. Further dilutions of each stock were prepared in a medium adapted to the requirements of the test. Since ethanol was used as initial solvent for ethanolic and acetonic bark extracts, finally reaching the highest concentration of 2.5% in chosen samples, two types of control sample were prepared for each experiment: bacterial/eukaryotic cells in medium alone (control 1) or in medium containing 2.5% ethanol (control 2). The reference compound, chlorogenic acid (SERVA Feinbiochemica, Heidelberg, Germany), was dissolved in 50% ethanol and diluted in the appropriate medium.

### 4.3. Cytotoxicity of V. opulus Fruit and Bark Extracts

A monolayer of human foreskin fibroblasts (HFF-1, ATCC-SCRC-104, LGC Standards, Łomianki, Poland) was cultured as previously described [26]. Briefly, a detached cell suspension (2.5 × 10^5^ cell/mL) was seeded (100 μL) into 96-well tissue culture plates (Nunc, Roskilde, Denmark) for 24 h at 37 °C/5%CO_2_. The culture medium was replaced with 100 μL of medium containing *V. opulus* extracts at a concentration range of 0.49–500 µg/mL for 24 h. Appropriate growth controls (the cells in culture medium alone and in medium containing 2.5% ethanol) were set up at the same time. The cytotoxic activity of the extracts was measured by MTT [3-(4,5-dimethylthiazole-2-yl)-2,5-diphenyltetrazolium bromide] reduction assay. After exposure, the medium containing extracts were removed, the cells were washed with phosphate-buffered saline (PBS; BioWest, Nuaillé, France), and then fresh cell culture (100 µL/well) and MTT (1.5 mg/mL, 50 µL/well) were added for 2 h of incubation under the above conditions. Finally, the MTT solution was removed and replaced with 75 μL/well of 20% sodium dodecyl sulfate (SDS; Sigma, St. Louis, MO, USA) for 24 h (room temperature) to dissolve the blue formazan produced by living cells. The absorbance of the samples was read at 550 nm with a microplate reader (Victor2, Wallac, Turku, Finland), and the percentage of viable cells was calculated based on the absorbance of appropriate growth controls considered to represent 100% cell viability. The experiment was carried out twice in duplicate each.

### 4.4. Staphylococcal Strains and Culture Conditions

The reference strains *S. aureus* ATCC 29213 (methicillin-sensitive *S. aureus*—MSSA) and *S. aureus* ATCC 43300 (methicillin-resistant *S. aureus*—MRSA), as well as the clinical isolate *S. aureus* H9 (MRSA), were used in the study. Bacteria from the stock suspensions kept frozen at –80 °C were grown for 24 h at 37 °C in tryptic soy agar (TSA; BTL Sp. z o.o., Łódź, Poland) to check the typical morphology and purity. Before setting up each experiment, cultures were freshly prepared in tryptic soy broth (TSB; BTL Sp. z o.o., Łódź, Poland) with 0.25% glucose (TSB/Glu) (for 24 h at 37 °C). The suspension density was adjusted to the appropriate value for the given test.

### 4.5. Minimal Inhibitory/Bactericidal Concentration (MIC/MBC)

The MICs of *V. opulus* extracts tested at a final concentration range of 15.6–2000 µg/mL against *S. aureus* ATCC 29213 were determined with a microdilution broth assay according to the EUCAST recommendations [71]. The MIC was defined as the lowest concentration of the extract that inhibited bacterial growth after 24 h of incubation at 37 °C compared to a positive growth control. If the MIC was established, 10 μL from the wells marked as having the MIC and from the wells having higher concentration or if the MIC was above the tested range, 10 μL from two wells with the highest concentration of the extract, were subcultured in TSA for 24 h at 37 °C to evaluate the MBC value. The MBC refers to the lowest concentration of the extract required to kill 99.9% of the bacteria.

### 4.6. SrtA Activity Testing

The effect of *V. opulus* extracts or chlorogenic acid (ChA; reference compound) used at 100 µg/mL and 500 µg/mL on SrtA activity was evaluated using SensoLyte 520 Sortase A Activity Assay Kit Fluorimetric (AnaSpec Inc., Fremont, CA, USA) in accordance with the manufacturer’s instructions. SrtA was exposed to the extracts/ChA for 10 min at 37 °C before substrate adding. Untreated SrtA as positive control and substrate alone was added. After 30 and 60 min ongoing incubation with the substrate the fluorescence of test and control samples at 490_ex_/520_em_ was read using a Spectra Max i3 (Molecular Devices, San Jose, CA, USA) in the Laboratory of Microscopic Imaging and Specialized Biological Techniques at the Faculty of Biology and Environmental Protection University of Lodz. The fluorescence reading from the substrate alone was used as a background and was subtracted from the fluorescence of the samples. The results are given as the percentage of SrtA activity calculated from relative fluorescence units (RFU) of test wells compared to the positive control of untreated SrtA, taken as 100% activity. The experiment was repeated twice.

### 4.7. Assessment of SpA Expression

The reference *S. aureus* ATCC 29213, *S. aureus* ATCC 43300, and clinical *S. aureus* H9 strain suspensions (OD_535_ = 0.5) were exposed to *V. opulus* extracts or ChA at concentrations of 100 µg/mL and 500 µg/mL (sample total volume 1 mL) for 24 h at 37 °C on an orbital shaker type TR-250/CH-4103 (Bottmingen, Switzerland). Bacterial suspensions in TSB/Glu medium alone served as the positive control. After incubation, bacteria were centrifuged at 3000 rpm for 10 min, washed twice with PBS (BioWest, Nuaillé, France) in a final volume of 5 mL, and finally, resuspended in 1 mL of PBS. To exclude the possible influence of a rinsing step on the number of bacterial cells, new suspensions were prepared at OD_535_ = 1.5. FITC-labelled goat polyclonal anti-SpA antibodies (Ab; GeneTex Inc., Irvine, CA, USA) diluted 1:100 in PBS with 1% bovine serum albumin (BSA; Sigma, St. Louis, MO, USA) were used for the detection of SpA expression on bacterial cells (the final Ab dilution 1:500). The SpA-related fluorescence was measured by Victor2 (Wallac, Turku, Finland), and based on relative fluorescence units (RFU), the percentage of SpA expression in comparison with that of the positive control (untreated bacteria taken as 100% SpA expression) was calculated. Two independent experiments were run for each strain in duplicate.

### 4.8. Extraction and Examination of the Composition of Glycolipids, Phospholipids, and Fatty Acids in Staphylococcal Membranes

#### 4.8.1. Chemicals

Standards of phospholipids were purchased from Avanti Polar Lipids (Alabaster, AL, USA). The other chemicals were acquired from Sigma-Aldrich (Darmstadt, Germany) and Avantor (Gliwice, Poland). All chemicals used were high-purity-grade reagents.

#### 4.8.2. Exposure of *S. aureus* to *V. opulus* Extracts

A suspension of *S. aureus* ATCC 43300 (OD_535_ = 1.8) in TSB/Glu prepared from fresh 24 h old culture was added (6 mL) to bacteriological Falcon-like tubes (Medlab, Raszyn, Poland) and centrifuged at 3000 rpm for 10 min. Bacteria in the sediment were then exposed to acetonic and ethanolic extracts from *V. opulus* fruits and bark at a concentration of 500 µg/mL (tested samples) for a 24 h at 37 °C with gentle shaking. Bacteria in culture medium alone (TSB/Glu) were prepared as control 1 and bacteria in culture medium TSB/Glu containing 2.5% ethanol were used as control 2. After exposure, the samples were centrifuged as described above, bacteria were suspended in 6 mL TSB/Glu, and each sample was added (2 mL) to three Eppendorf tubes (Medlab, Raszyn, Poland) for lipid and fatty acid extraction and analysis. The whole experiment was conducted twice.

#### 4.8.3. Extraction of the Lipids

Lipids from *S. aureus* cells were extracted according to our previous method [25] with modifications. The bacterial biomass was moved into 2 mL Eppendorf tubes containing glass beads, 0.33 mL methanol, and 0.66 mL chloroform. Then, the samples were homogenized for 1 min with the use of FastPrep (MP Biomedicals, Shanghai, China). After, the samples had been centrifuged (2000× *g*, 2 min), the supernatants were transferred into 1.5 mL Eppendorf tubes, and 0.2 mL of saline was added. The lower layer of each sample was collected and evaporated. The obtained extracts were dissolved in 0.75 mL methanol.

#### 4.8.4. Staphylococcal Lipid Analysis

The lipid content was determined using an Agilent 1200 HPLC (Santa Clara, CA, USA) and a 4500 QTRAP mass spectrometer (Sciex, Framingham, MA, USA) equipped with an ESI source operated in positive or negative ion mode. Ten microliters of the lipid extract were injected onto a Kinetex C18 column (50 mm × 2.1 mm, particle size: 5 μm; Phenomenex, Torrance, CA, USA) at a flow rate of 0.5 mL/min and a temperature of 40 °C. The gradient elution was applied with mobile phases of water (A) and methanol (B) (both consisted of 5 mM ammonium formate). The solvent gradient was initiated at 70% B, increased to 95% B over 1.25 min, and maintained at 95% B for 6 min before returning to the initial solvent composition over 3 min. The data analysis was performed with Analyst™ v1.6.2 software (Sciex, Framingham, MA, USA).

At first, the untargeted approach was performed with precursor ion scanning at m/z 153 (in negative mode) or neutral loss scanning of *m*/*z* 300 (in positive ion mode) to detect PG and Lysyl-PG, respectively, triggering enhanced product ion experiments. Sodium adducts of glycolipids were searched using enhanced mass spec experiments. On the basis of the untargeted analysis, a comprehensive list of the multiple reaction monitoring (MRM) transitions was generated, and a quantitative analysis was performed.

#### 4.8.5. Staphylococcal Fatty Acid Analysis

To Eppendorfs with bacterial lipid extract (0.375 mL), toluene (0.05 mL) and 8.0% HCl solution in methanol (0.075 mL) were added [72]. The tubes were vortexed and then incubated at 45 °C for 16 h. After cooling to room temperature, 0.5 mL hexane and 0.5 mL water were added for the extraction of fatty acid methyl esters (FAMEs). One microliter of each extract sample was analyzed using a gas chromatography system (Agilent Model 7890 gas chromatograph, equipped with a 5975C mass detector). The separation was carried out in a HP 5 MS methyl polysiloxane capillary column (30 m × 0.25 mm i.d. × 0.25 mm ft). The column temperature was maintained at 60 °C for 3 min, and then increased to 212 °C at a rate of 6 °C/min, followed by an increase to 245 °C at a rate of 2 °C/min, and finally, to 280 °C at a rate of 20 °C/min for 10 min. Helium was used as the carrier gas at a flow rate of 1 mL/min. The injection port temperature was 250 °C. Bacterial fatty acids were identified by comparison with the retention times of the authentic standards (Sigma-Aldrich, Darmstadt, Germany), and the results are expressed as a percentage of the total amount of fatty acids.

### 4.9. Assessment of Biofilm Formation by S. aureus

The suspensions of all tested bacteria at a density of OD_535_ = 0.5 in TSB/Glu were exposed to *V. opulus* extracts or chlorogenic acid (reference compound) at final concentrations of 100 and 500 µg/mL (sample total volume 1 mL) for 24 h at 37 °C on an orbital shaker (TR-250/CH-4103, Bottmingen, Switzerland). Control bacteria were incubated in TSB/Glu alone. After incubation, the bacteria were harvested by centrifugation at 3000 rpm/10 min, washed twice with PBS in a volume of 5 mL, and finally resuspended in 1 mL of TSB/Glu. To exclude possible influence of a rinsing step on the number of bacterial cells, new suspensions at a density of OD_535_ = 0.9 were prepared in TSB/Glu and seeded (100 μL) into the wells of 96-well polystyrene culture microtiter plates (Nunc, Denmark) for 24 h at 37 °C to develop a biofilm. Proper negative control (medium alone) and positive controls (bacteria untreated with the extracts) were included. The effects of *V. opulus* extracts were assessed with the LIVE/DEAD BacLight Bacterial Viability Kit (Invitrogen, Molecular Probes, Eugene, OR, USA) according to the manufacturer’s recommendations. Finally, the fluorescence of the samples was measured at 485_ex_/535_em_ nm for green Syto-9 and 485_ex_/620_em_ for red PI using a Spectra Max i3 (Molecular Devices, San Jose, CA, USA) in the Laboratory of Microscopic Imaging and Specialized Biological Techniques at the Faculty of Biology and Environmental Protection University of Lodz. Each variant used for the experiment was tested twice in quadruplicate. The results are given as the percentage of biofilm biomass calculated from the mean fluorescence values ± SD of test wells in comparison with the positive control (untreated bacteria, taken as 100% biofilm).

### 4.10. Statistics

Data are expressed as the mean ± standard deviation (SD). The differences in biochemical composition of the extracts were analyzed using one-way analysis of variance (ANOVA) and Tukey’s test. The differences in biological activity of the extracts were evaluated using the Mann–Whitney *U* test and one-way ANOVA. STATISTICA 13.1 (StatSoft Polska Sp. Z o.o., Kraków, Poland) software was used for the calculations. Differences with *p* ≤ 0.05 were considered statistically significant.

## 5. Conclusions

In summary, the consumption of *V. opulus* fruit and bark extracts may be beneficial for the prevention of staphylococcal infections due to their negative impacts on some *S. aureus* virulence factors, such as SrtA activity, SpA expression, and cell membrane modification conditioning proper homeostasis and increased resistance. These extracts may also limit biofilm formation by staphylococci. Nevertheless, full inhibition of the development of these structures cannot be assumed using *V. opulus* extracts as the only preventive preparations. Although we have recognized several limitations of the present study, the results obtained reveal novel insights regarding the pro-health use of *V. opulus* extracts to prevent staphylococcal infections.

## Figures and Tables

**Table 1:** Chemical composition of *V. opulus* fruit and bark extracts.

Components	Extraction Solvent	Fruit (VF)	Bark (VB)
Protein (mg/g)	70% acetone	11.38 ± 0.88 Aa	13.14 ± 0.90 Ab
	70% ethanol	9.63 ± 0.88 Aa	8.64 ± 0.01 Aa
	water	14.29 ± 0.15 Ab	16.13 ± 0.93 Bc
Sugars (mg/g)	70% acetone	606.87 ± 9.97 Bb	186.00 ± 0.52 Ab
	70% ethanol	674.69 ± 18.67 Bc	192.95 ± 1.45 Ac
	water	541.79 ± 30.58 Ba	162.38 ± 2.18 Aa
Organic acids (mg/g)	70% acetone	79.05 ± 1.24 Bb	6.26 ± 0.21 Ab
	70% ethanol	86.36 ± 0.48 Bc	7.24 ± 0.04 Ac
	water	71.51 ± 4.89 Ba	4.26 ± 0.09 Aa
Total phenolics ^1^ (mg GAE/g)	70% acetone	80.47 ± 3.86 Ac	254.97 ± 1.54 Bc
	70% ethanol	71.76 ± 3.02 Ab	218.53 ± 2.07 Bb
	water	50.64 ± 1.53 Aa	171.02 ± 3.16 Ba
Flavanols ^2^ (mg CE/g)	70% acetone	18.58 ± 0.19 Ac	91.51 ± 2.38 Ba
	70% ethanol	14.44 ± 0.73 Ab	96.69 ± 2.59 Bab
	water	11.82 ± 1.19 Aa	97.79 ± 3.71 Bb
Proanthocyanidins (mg CYE/g)	70% acetone	12.26 ± 0.46 Ac	63.83 ± 4.60 Bc
	70% ethanol	10.06 ± 0.46 Ab	56.67 ± 1.41 Bb
	water	5.81 ± 0.31 Aa	50.29 ± 1.80 Ba
Total hydroxycinnamic acids ^4^ (mg CA/g)	70% acetone	65.04 ± 0.49 Bb	26.20 ± 0.02 Ab
	70% ethanol	65.64 ± 0.29 Bb	26.65 ± 0.01 Ac
	water	60.37 ± 0.04 ba	14.24 ± 0.02 Aa
Total flavanols^4^ (mg CE/g)	70% acetone	11.58 ± 0.07 Ac	132.15 ± 0.13 Bc
	70% ethanol	10.33 ± 0.07 Ab	127.25 ± 0.12 Bb
	water	8.84 ± 0.02 Aa	119.13 ± 0.08 Ba
Total flavonols ^4^ (mg QG/g)	70% acetone	0.56 ± 0.01 b	-
	70% ethanol	0.61 ± 0.01 c	-
	water	0.48 ± 0.01 a	-
Total flavalignans ^4^ (mg CIN/g)	70% acetone	1.13 ± 0.03 Aa	3.93 ± 0.03 Ba
	70% ethanol	1.92 ± 0.09 Ab	4.07 ± 0.10 Ba
	water	4.30 ± 0.04 Ac	6.04 ± 0.01 Bb
Total iridoids ^4^ (mg QR/g)	70% acetone	-	15.05 ± 0.23 a
	70% ethanol	-	15.44 ± 0.19 a
	water	-	23.66 ± 0.51 b

Values are presented as the means ± SD, *n* = 3, ^1^ determined by the Folin–Ciocalteu method; ^2^ determined by the vanillin method; ^3^ determined after acid depolymerisation; ^4^ determined by UPLC analysis; GAE—gallic acid equivalents; CE—(+)-catechin equivalents; CYE—cyanidin equivalents; CA—chlorogenic acid equivalents; QG—quercetin 3-glucoside equivalents; CIN—cinchonine equivalents; QR—quercetin 3-rutinoside equivalents. The means within the same column with different letters (a, b, c) or within the same row with different letters (A, B, C) are significantly different according to Tukey’s test (*p* ≤ 0.05).

**Table 2:** MIC/MBC of the extracts from *V. opulus* fruits and bark, and activity of chlorogenic acid against *S. aureus* ATCC 29213.

Concentration [mg/mL]	VFa	VFe	VFw	VBa	VBe	VBw	ChA
MIC	2	2	>2	1	2	2	>2
MBC	>2	2	>2	1	>2	>2	>2

MIC—minimal inhibitory concentration; MBC—minimal bactericidal concentration; VF—*V. opulus* fruit extract; VB—*V. opulus* bark extract; a/e/w—acetonic/ethanolic/water extract; ChA—chlorogenic acid.

## Data Availability

The data presented in this study are available on request from the corresponding author.

## Supplementary Materials

The following are available online. Figure S1: UPLC chromatograms of phenolic compounds in *V. opulus* fruit or bark ethanolic extract at 280 nm, Figure S2: The effects of *V. opulus* extracts on the contents of specific types of lipids in the cell membrane of *S. aureus* ATCC 43300: (a) PG—phosphatidylglycerols; (b) LYSYL-PG—lysyl-phosphatidylglycerols; (c) GLC2-DAG—diglucosyldiacylglycerols, Figure S3: The effects of *V. opulus* extracts on the contents of specific types of fatty acids in the cell membrane of *S. aureus* ATCC 43300, Table S1: Characterization of phenolic compounds in different *V. opulus* fruit and bark extracts by LC-QTOF-MS analysis in negative ion mode.

## Sample Availability

Samples of the compounds are not available from the authors.
