# Supplementary material for: An In Vitro Study of the Effect of Viburnum opulus Extracts on Key Processes in the Development of Staphylococcal Infections
Source: Molecules. 2021 Mar 21;26(6):1758. doi: 10.3390/molecules26061758 (PMC8003844; doi:10.3390/molecules26061758)
Supplement: Supplementary file 1 [file molecules-26-01758-s001.pdf]

SUPPLEMENTARY MATERIALS

An in vitro study of the effect of *Viburnum opulus* extracts on key processes in the development of staphylococcal infections

Urszula Wójcik-Bojek[1], Joanna Rywaniak[1], Przemysław Bernat[2], Anna Podsędek[3], Dominika Kajszczak[3], Beata Sadowska[1,*]

1 Department of Immunology and Infectious Biology, Institute of Microbiology, Biotechnology and Immunology, Faculty of Biology and Environmental Protection, University of Lodz, Banacha 12/16, 90-237 Lodz, Poland; urszula.wojcik@biol.uni.lodz.pl (U.W.-B.); joanna.rywaniak@biol.uni.lodz.pl (J.R.); beata.sadowska@biol.uni.lodz.pl (B.S.)

2 Department of Industrial Microbiology and Biotechnology, Institute of Microbiology, Biotechnology and Immunology, Faculty of Biology and Environmental Protection, University of Lodz, Banacha 12/16, 90-237 Lodz, Poland; przemyslaw.bernat@biol.uni.lodz.pl

3 Institute of Molecular and Industrial Biotechnology, Faculty of Biotechnology and Food Sciences, Lodz University of Technology, Stefanowskiego 4/10, 90-924 Lodz, Poland; anna.podsedek@p.lodz.pl (A.P.); dominika.kajszczak@dokt.p.lodz.pl (D.K.)

Correspondence: beata.sadowska@biol.uni.lodz.pl; Tel.: +48 42 635 45 25 (B.S.)

2. Results

2.1. Biochemical characterization of V. opulus fruit and bark extracts

Qualitative analysis of the *V. opulus* fruit and bark extracts by the UPLC-QTOF-MS technique revealed that the phenolic compound composition differed notably (Table S1 and Figure S1). It has been observed that bark phenols include hydroxycinnamic acids (peaks 1, 11, 12, 27, 30), flavanols (monomeric and oligomeric forms; peaks 4, 6, 8, 10, 14-16, 19), and flavalignans (peaks 18, 22), while fruit phenols include hydroxycinnamic acids (peaks 1-3, 5, 7, 9, 11-13, 17, 20), flavalignans (peaks 25, 26, 28), flavanols (peaks 15, 19, 31), and flavonols (peaks 21, 23, 24, 29). Additionally, based on molecular ions in the MS spectra and fragmentation patterns published by Dienaitė et al. [1], a viburtinoside derivative was identified in bark extracts.

Table S1. Characterization of phenolic compounds in different *V. opulus* fruit and bark extracts by LC-QTOF-MS analysis in negative ion mode.

No	R_t (min)	λ_{max} (nm)	$[M-H]^-$ (m/z)	MS/MS (m/z)	Identification	Fruit			Bark		
						VFa	VFe	VFw	VBa	VBe	VBw
1	4.11	324	353	191,135	Neochlorogenic acid[s]	+	+	+	+	+	+
2	4.18	326	515	191,135	Dicaffeoylquinic acid I[r]	+	+	+	-	-	-
3	4.60	325	515	191,135	Dicaffeoylquinic acid II[r]	+	+	+	-	-	-
4	4.70	279	577	125,161,255	Procyanidin B1[s]	-	-	-	+	+	+
5	5.02	326	515	191,135	Dicaffeoylquinic acid III[r]	+	+	+	-	-	-

6	5.23	281	865	407,289,125	Procyanidin trimer I[r]	-	-	-	+	+	+
7	5.27	326	515	191,135	Dicaffeoylquinic acid IV[r]	+	+	+	-	-	-
8	5.35	278	289	109,159,173	(+)-Catechin[s]	-	-	-	+	+	+
9	5.38	326	515	191,135	Dicaffeoylquinic acid V[r]	+	+	+	-	-	-
10	5.54	279	865	407,289,125	Procyanidin trimer II[r]	-	-	-	+	+	+
11	5.78	325	353	191,133	Chlorogenic acid[s]	+	+	+	+	+	+
12	5.97	326	353	191,135	Cryptochlorogenic acid[s]	+	+	+	+	+	+
13	6.15	324	179	132,108	Caffeic acid[s]	+	+	+	-	-	-
14	6.17	279	577	125,161,203, 255	Procyanidin B2[s]	-	-	-	+	+	+
15	6.55	341	1153	287,407,125, 243	Procyanidin tetramer[r]	+	+	+	+	+	-
16	6.99	279	289	109,159,173	(-)-Epicatechin[s]	-	-	-	+	+	+
17	7.31	311	337	119,191	Coumaroylquinic acid[r]	+	+	+	-	-	-
18	7.50	279	739	177,289,161, 245	Cinchonain IIx[r]	-	-	-	+	+	+
19	7.62	278	865	407,289,125	Procyanidin C1[s]	+	+	+	+	+	+
20	9.03	326	367	135	Feruoylquinic acid[r]	+	+	-	-	-	-
21	9.21	354	595	271,300,255, 243	Quercetin pentoside hexoside[s]	+	+	+	-	-	-
22	9.32	279	451	176,191,269	Cinchonain Ix[r]	-	-	-	+	+	+
23	9.81	352	609	271,300,255, 243	Quercetin 3-rutinoside[s]	+	+	+	-	-	-
24	10.10	343	463	271,255,243, 227	Quercetin 3-glucoside[s]	+	+	+	-	-	-
25	10.77	343	451	189,161	Cinchonin Ix[r]	+	+	+	-	-	-
26	10.85	283	451	189,161	Cinchonin Ix[r]	+	+	+	-	-	-
27	10.87	326	515	191,135	3,5-Dicaffeoylquinic acid[s]	-	-	-	+	+	+
28	10.95	281	451	189,161	Cinchonin Ix[r]	+	+	+	-	-	-
29	11.33	343	447	227,255,183, 299	Quercetin 3-rhamnoside[s]	+	+	+	-	-	-
30	11.82	326	515	191,135	Dicaffeoylquinic acid VI[r]	-	-	-	+	+	+
31	12.94	279	613	242,393,189, 309	(Epi)-catechin hexoside[r]	+	+	+	-	-	-
32	12.95	281	607	231,339,181	Viburtinoside derivative[r]	-	-	-	+	+	+

+ compound present in extract; - compound not detected; superscript letters with the compound name: ^{s} confirmed by a standard; ^{r} confirmed by a reference [1–3]; VF—*V. opulus* fruit extract; VB—*V. opulus* bark extract; a/e/w—acetonic/ethanolic/water extract.

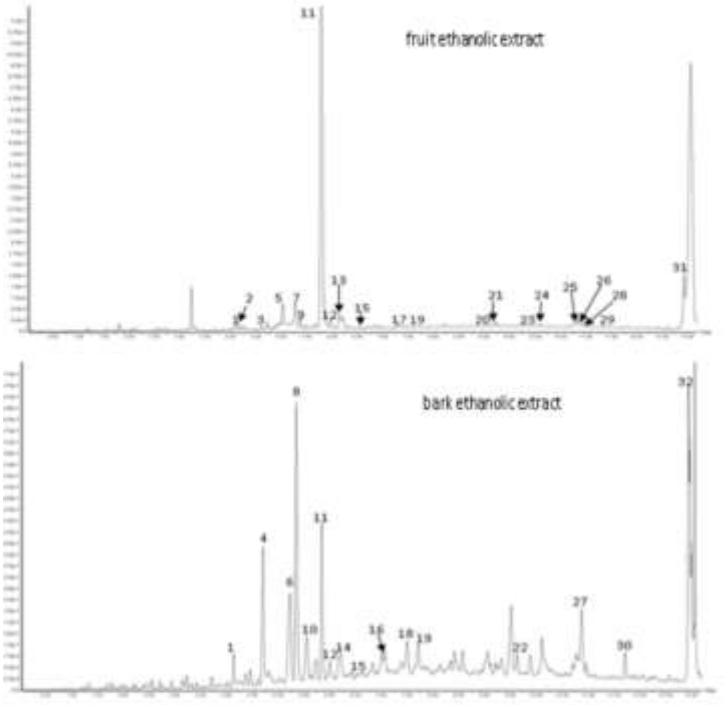

Figure S1. UPLC chromatograms of phenolic compounds in *V. opulus* fruit or bark ethanolic extract at 280 nm. Refer to Table S1 for the identification of each numbered peak.

2.6. The effects of the tested extracts on the compositions of phospholipids and fatty acids in staphylococcal membranes

The analysis of the changes in content of specific types of lipids (Figure S2) showed pronounced rises in PG 18:0/15:0, PG 16:0/15:0, and PG 15:0/14:0 (Figure S2a) as well as GLC2-DAG 17:0/15:0 and GLC2-DAG 16:0/15:0 (Figure S2b) after VBa and VBe treatment. Under the same conditions, drops in content of LYSYL-PG 20:0/15:0, LYSYL-PG 19:0/15:0, and LYSYL-PG 17:0/15:0 were detected (Figure S2c). On the other hand, decreases in PG 19:0/15:0 and GLC2-DAG 19:0/15:0 and an increase in LYSYL-PG 16:0/15:0 were also noted but with no influence on general changes to the percentage contents of these phospholipid groups. Otherwise, there were no significant differences in the level of branched fatty acids (BCFAs) with isomerism in terms of both iso (C20:0, C18:0, C16:0, and C14:0) and anteiso (aiC15:0) compounds in the membranes of *S. aureus* exposed to *V. opulus* extracts in comparison with control cells (Figure S3).

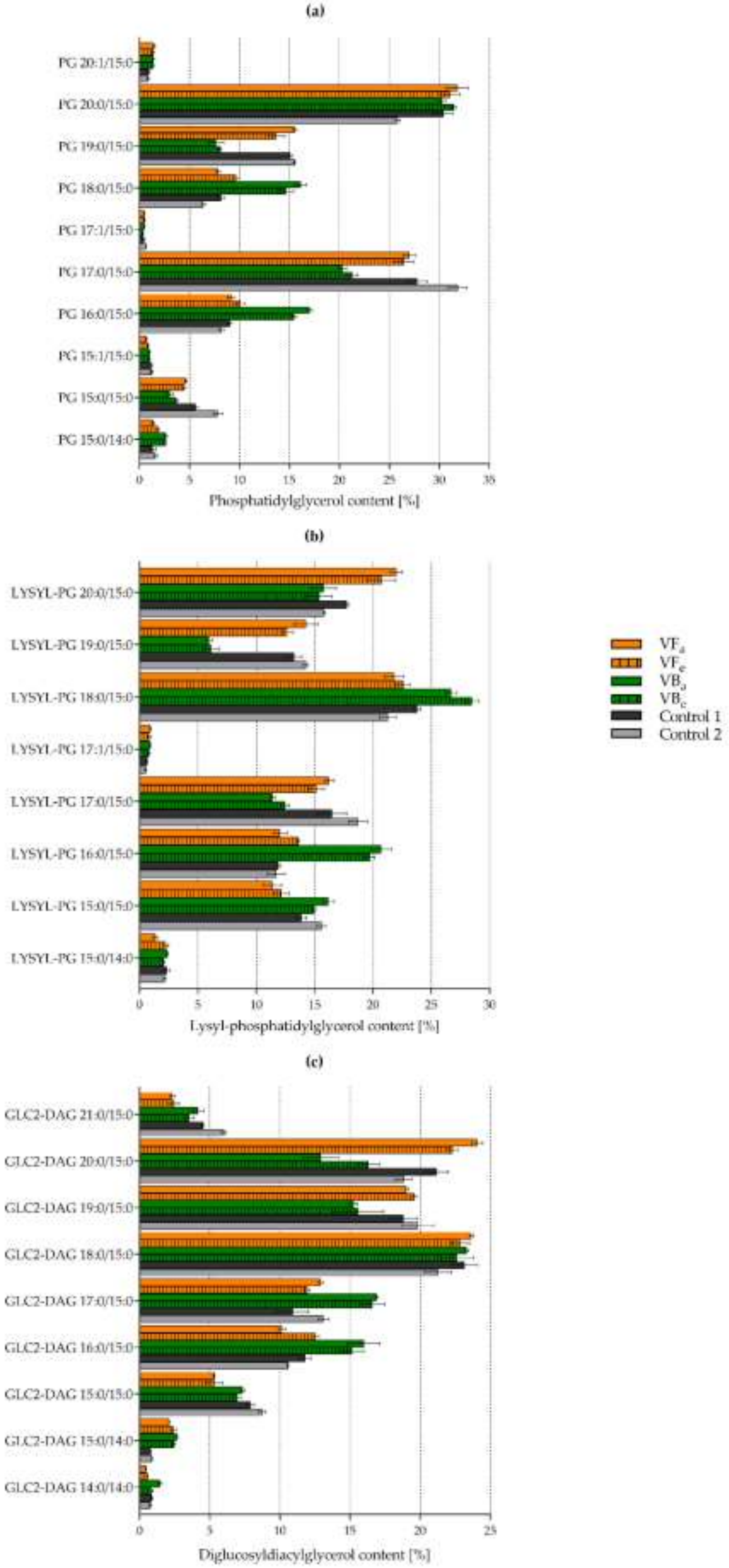
(a)
PG 20:1/15:0
PG 20:0/15:0
PG 19:0/15:0
PG 18:0/15:0
PG 17:1/15:0
PG 17:0/15:0
PG 16:0/15:0
PG 15:1/15:0
PG 15:0/15:0
PG 15:0/14:0
0 5 10 15 20 25 30 35
Phosphatidylglycerol content [%]
(b)
LYSYL-PG 20:0/15:0
LYSYL-PG 19:0/15:0
LYSYL-PG 18:0/15:0
LYSYL-PG 17:1/15:0
LYSYL-PG 17:0/15:0
LYSYL-PG 16:0/15:0
LYSYL-PG 15:0/15:0
LYSYL-PG 15:0/14:0
0 5 10 15 20 25 30
Lysyl-phosphatidylglycerol content [%]
(c)
GLC2-DAG 21:0/15:0
GLC2-DAG 20:0/15:0
GLC2-DAG 19:0/15:0
GLC2-DAG 18:0/15:0
GLC2-DAG 17:0/15:0
GLC2-DAG 16:0/15:0
GLC2-DAG 15:0/15:0
GLC2-DAG 15:0/14:0
GLC2-DAG 14:0/14:0
0 5 10 15 20 25
Diglucosyldiacylglycerol content [%]
VF_a
VF_e
VB_a
VB_e
Control 1
Control 2

Figure S2. The effects of *V. opulus* extracts on the contents of specific types of lipids in the cell membrane of *S. aureus* ATCC 43300: (a) PG—phosphatidylglycerols; (b) LYSYL-PG—lysyl-phosphatidylglycerols; (c) GLC2-DAG—diglucosyldiacylglycerols; VF—*V. opulus* fruit extract; VB—*V. opulus* bark extract; a/e—acetonic/ethanolic extract; Control 1—bacteria in medium alone; Control 2—bacteria in medium containing 2.5% ethanol.

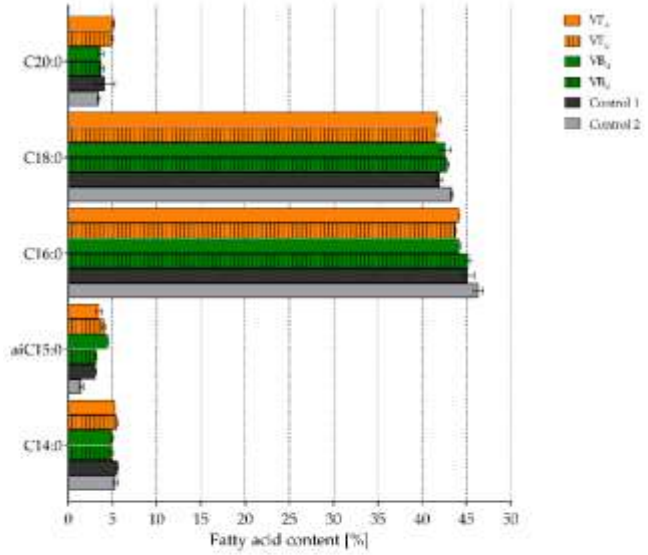

Figure S3. The effects of *V. opulus* extracts on the contents of specific types of fatty acids in the cell membrane of *S. aureus* ATCC 43300; VF—*V. opulus* fruit extract; VB—*V. opulus* bark extract; a/e—acetonic/ethanolic extract; Control 1—bacteria in medium alone; Control 2—bacteria in medium containing 2.5% ethanol.

5. References

1. Dienaitė, L.; Pukalskienė, M.; Pereira, C.V.; Matias, A.A.; Venskutonis, P.R. Valorization of European cranberry bush (*Viburnum opulus* L.) berry pomace extracts isolated with pressurized ethanol and water by assessing their phytochemical composition, antioxidant, and antiproliferative activities. *Foods* **2020**, *9*, 1413, doi:10.3390/foods9101413.
2. Senica, M.; Stampar, F.; Veberic, R.; Mikulic-Petkovsek, M. Fruit seeds of the *Rosaceae* family: a waste, new life, or a danger to human health? *J. Agric. Food Chem.* **2017**, *65*, 10621–10629, doi:10.1021/acs.jafc.7b03408.
3. Wojdyło, A.; Oszmiański, J.; Bielicki, P. Polyphenolic composition, antioxidant activity, and polyphenol oxidase (PPO) activity of quince (*Cydonia oblonga* Miller) varieties. *J. Agric. Food Chem.* **2013**, *61*, 2762–2772, doi:10.1021/jf304969b.
